# A20 suppresses hepatocellular carcinoma proliferation and metastasis through inhibition of Twist1 expression

**DOI:** 10.1186/s12943-015-0454-6

**Published:** 2015-11-04

**Authors:** Haiyang Chen, Liang Hu, Zaili Luo, Jian Zhang, Cunzhen Zhang, Bijun Qiu, Liwei Dong, Yexiong Tan, Jin Ding, Shanhua Tang, Feng Shen, Zhong Li, Hongyang Wang

**Affiliations:** International Cooperation Laboratory on Signal Transduction, Eastern Hepatobiliary Surgery Institute/Hospital, The Second Military Medical University, 225 Changhai Road, Shanghai, 200438 China; Anal-Colorectal Surgery Institute, 150th Hospital of PLA, Luoyang, China; Shanghai Institute of Cell Therapy Research, Shanghai, China; National Center for Liver Cancer, Shanghai, China

**Keywords:** A20, Hepatocellular carcinoma, Proliferation, Metastasis, Twist 1

## Abstract

**Background:**

Aberrant expression of A20 has been reported in several human malignancies including hepatocellular carcinoma (HCC). However, its clinical relevance and potential role in HCC remain unknown.

**Methods:**

Quantitative PCR, Western blots and immunohistochemistry analyses were used to quantify A20 expression in HCC samples and cell lines. The correlation of A20 expression with clinicopathologic features was analyzed in a cohort containing 143 patients with primary HCC. Kaplan-Meier curves were used to evaluate the association between A20 expression and patient survival. Functional studies were performed to determine the effects of A20 on proliferation and metastasis of HCC cells in vitro and in vivo.

**Results:**

Expression of A20 was increased in HCC tissues and cell lines. Increased expression of A20 was negatively correlated with the tumor size, TNM stage, tumor thrombus formation, capsular invasion and serum AFP levels. Patients with higher A20 expression had a prolonged disease-free survival and overall survival than those with lower A20 expression. Forced expression of A20 significantly inhibited the proliferative and invasive properties of HCC cells both in vitro and in vivo, whereas knockdown of A20 expression showed the opposite effects. Further studies revealed that expression of A20 was inversely correlated with Twist1 levels and NF-κB activity in HCC tissues and cell lines. A20-induced suppression of proliferation and migration of HCC cells were mainly mediated through inhibition of Twist1 expression that was regulated at least partly by A20-induced attenuation of NF-κB activity.

**Conclusions:**

Our results demonstrate that A20 plays a negative role in the development and progression of HCC probably through inhibiting Twist1 expression. A20 may serve as a novel prognostic biomarker and potential therapeutic target for HCC patients.

**Electronic supplementary material:**

The online version of this article (doi:10.1186/s12943-015-0454-6) contains supplementary material, which is available to authorized users.

## Introduction

Liver cancer is one of the leading causes of cancer-related death worldwide and is particularly prevalent in China. According to data from the International Agency for Research on Cancer, there were an estimated 782,500 new cases of liver cancer and 745,500 deaths during 2012, with China alone accounting for about 50 % of the total number of cases and deaths [1]. Among primary liver cancers, hepatocellular carcinoma (HCC) represents the major histological subtype, accounting for 70–85 % of the total liver cancer burden worldwide [2]. Despite the advances in diagnosis and treatment, the prognosis of HCC patients remains dismal due to the high frequency of tumor recurrence or distant metastasis after surgical resection [3]. Therefore, a better understanding of the molecular mechanisms involved in the pathogenesis and progression of HCC is particularly important for the development of novel therapeutic strategies for the treatment of patients with HCC.

A20, also known as Tumor necrosis factor α-induced protein 3 (TNFAIP3), was initially identified as a primary gene product following tumor necrosis factor α (TNFα) treatment in human umbilical vein endothelial cells [4]. Previous studies of the A20 gene have focused mainly on the immunoregulatory aspects. It has been shown that A20 is a central regulator of cellular immune and inflammation and acts as an ubiquitin-editing enzyme to potently downregulate NF-κB signaling, thereby functioning as an important anti-inflammatory factor [5]. Recently, aberrant expression of A20 has been reported in several human malignancies and growing evidence supports the notion that A20 also plays a functional role in cancer development. It has been identified that A20 is a crucial tumor suppressor in various lymphomas, such as diffuse large B-cell lymphoma, classical Hodgkin's lymphoma, mucosa-associated tissue lymphoma and the T-cell malignancy-Sezary syndrome SS [6–10]. In addition, reduced expression of A20 was also observed in pancreatic cancer tissues [11]. In contrast to lymphoma and pancreatic cancer, A20 is highly expressed and responsible for proliferation of glioblastomas and bladder cancer [12–15]. These discrepant reports on the role of A20 in different malignances indicate that its biological function is tissue-dependent and varies with the type of malignancy [16]. In liver, A20 was mainly known as a promoter of proliferation based on hepatectomy and liver regeneration experiments in A20 knock-out mice [17]. Interestingly, Dong et al. recently reported that A20 was highly expressed in human HCC tissues [18]. Given the potential significance of A20 in cancer pathobiology, its clinical relevance and potential role in human HCC deserves to be investigated.

Therefore, in the present study, we examined both the mRNA and protein expression levels of A20 in HCC tissue samples and cell lines and further analyzed the clinical significance of A20 expression in a cohort of HCC patients. Moreover, we explored the potential role of A20 in HCC cell proliferation and migration both in vitro *and* in vivo. Our findings may shed a new light on the pathogenesis of HCC and provide a novel therapeutic target for the treatment of patients with HCC.

## Materials and methods

### Patients and follow-up

Formalin-fixed paraffin-embedded tissue specimens from 143 primary HCC patients who received curative surgery in the Eastern Hepatobiliary Surgery Hospital (Shanghai, China) from September 2008 to June 2010 were retrieved for immunohistochemistry. Detailed clinicopathologic characteristics of the patients are listed in Table 1. The follow-up period was defined as the interval from the date of surgery to the date of death or last follow-up. The latest follow-up was updated in September 2013. Overall survival (OS) was defined as the interval from the date of surgery to the date of death. Patients alive at the end of follow-up were censored. Disease-free survival (DFS) was defined as the interval from the date of surgery to the date of disease recurrence; if recurrence was not diagnosed, patients were censored on the date of death or last follow-up. Patients were excluded from the study cohorts with the following exclusion criteria: previously received any anticancer therapy; impaired heart, lung, liver or kidney function; previous malignant disease. Tumor stage was classified according to the 7th Edition tumor-node-metastasis (TNM) classification of the American Joint Committee on Cancer Staging. Fresh-frozen HCC samples obtained from 84 primary HCC patients who received curative surgery in the Eastern Hepatobiliary Surgery Hospital from October 2012 to July 2013 were used for quantitative polymerase chain reaction (qPCR) and Western blot analysis. Written informed consent was obtained from each patient and this study was approved by the Ethics Boards of the Eastern Hepatobiliary Surgery Hospital.

**Table 1:** Relationship between Intratumor A20 expression and clinicopathologic features of HCC patients in the study cohort

A20 expression (*n* = 143)			
Variable	Low (*n* = 81)	High (*n* = 62)	*p* value ^**a**^
Sex			0.847
Male	71	55	
female	10	7	
Age (years)			0.185
≤ 50	52	33	
> 50	29	29	
Tumor size (cm)			**0.007**
≤ 5	14	23	
> 5	67	39	
HBsAg			0.078
Negative	3	7	
Positive	78	55	
Liver cirrhosis			0.389
No	27	25	
Yes	54	37	
TNM stage			**<0.001**
I,II	26	40	
III,IV	55	22	
Capsular formation			0.062
No	48	27	
Yes	33	35	
Thrombus formation			**0.002**
No	32	41	
Yes	49	21	
Capsular invasion			**0.038**
No	9	15	
Yes	72	47	
AFP			**0.013**
≤ 20U/L	13	21	
> 20U/L	68	41	

^a^ Pearson chi-square test was used for comparison between subgroups. Bold type indicates statistical significance

### Plasmids and biological reagents

pEF1-A20-wt was a gift from Dr Daniel Krappmann (Helmholtz Zentrum Munchen Gmbh, German). The pCSII-H1-PGK- puro-WPRE-shRNA-A20 and control scramble vector were kindly provided by Prof. Masao Seto. pBabe-puro-flag-twist1 was kindly provided by Prof. Alain Puisieux. Lentivirus vector pCDH-CMV-EF1-GFP-puro purchased from System Biosciences was constructed for A20 stable expression. The IκBα plasmid and the NFκB promoter-luciferase plasmid were purchased from the Addgene.

### Cell lines and culture

HCCLM3 cells were transferred from the cell bank of Zhongshan Hospital, Fudan University Medical College in 2012. HCCLM3 was established in 2003 in Zhongshan Hospital [19] and stored in liquid nitrogen tank. Normal liver cell lines QSG-7701 and liver cancer cell lines SMMC-7721, MHCC-97 L and MHCC-97H were purchased from the Cell Research Institute of Chinese Academy of Sciences (Shanghai, China). Cells were maintained at 37 °C in a humidified incubator containing 5 % CO_2_ in Dulbecco’s modified Eagle’s medium supplemented with 10 % heat-inactivated fetal bovine serum and passed every 2–3 d to maintain logarithmic growth. Stable knockdown or overexpression HCCLM3 cells (LM3-shA20 and LM3-A20, respectively) and their empty vector counterparts (LM3-shcon and LM3-con, respectively) were generated using a lentivirus system followed by selected in medium containing 3 μg/ml puromycin for 2–3 weeks.

### Tissue microarray and immunohistochemistry

Tissue microarrays (TMAs) containing the specimens from the Eastern Hepatobiliary Surgery Hospital were constructed (in collaboration with Shanghai Biochip Company, Shanghai, China). Immunohistochemistry of tissue microarray slides was performed as described previously [20]. Briefly, slides were deparaffinized and rehydrated. The endogenous peroxidase activity was blocked with 3 % H_2_O_2_ for 10 min. Antigens were retrieved with citrate buffer (10 mM, pH 6.0) for 15 min at 100 °C in a microwave oven. After blocking, the slides were incubated with a primary anti-A20 antibody (Santa Cruz, 1:50) or a primary anti-PCNA antibody (CST, 1:4000) at 4 °C overnight in a moist chamber followed by incubated with an anti-rabbit or anti-mouse peroxidase-conjugated secondary antibody (Santa Cruz) at room temperature for 30 min. Finally, the visualization signal was developed with 3,3′-diaminobenzidine (Dako) and the slides were counterstained with hematoxylin. Expression of A20 in the tissue chip was evaluated in a blinded manner without prior knowledge of the clinical data using the German immunoreactive score (IRS) [21]. The median value of the IRS was chosen as the cut-off for high and low A20 expression levels based on a measure of heterogeneity according to the log-rank test with respect to the overall survival as described previously [22, 23].

### Real-Time PCR

Real-time qPCR analysis was performed as described previously [20]. Briefly, Total RNA was prepared using Trizol (Invitrogen) from liver tumor and non-tumor tissues, and reversely transcribed into cDNA using M-MLV Reverse Transcriptase (Promega). Gene primers were ordered from Sangon Biotech (Shanghai) and SYBR Green PCR Master Mix was purchased from Takara Biotechonology Corporation. qPCR was performed on ABI Prism 7300 Sequence Detection System (Applied Biosystems) using the 2^-ΔΔCT^ method. Gene expression results were normalized by internal control β-actin. Primers used in this study were listed in Additional file 1: Table S1. Each sample was tested in triplicate.

### Western blots

Western blot assay was performed as described previously [20]. Briefly, tumor specimens were prepared in lysis buffer [Tris-HCl (20 mM), pH 7.4, NaCl (150 mM), glycerol (10 %), Nonidet P-40 (0.2 %), EDTA (1 mM), EGTA (1 mM), PMSF (1 mM), NaF (10 mM), aprotinin (5 mg/ml), leupeptin (20 mM), and sodium orthovanadate (1 mM)] and centrifuged at 12,000 g for 30 min. Protein concentrations were measured using the BCA assay. All samples were prepared to the same concentration with 4XSDS sample buffer. The proteins were separated by SDS-PAGE and transferred to nitrocellulose membranes. The primary antibodies specific for A20, Twist1 and β-actin were purchased from Santa Cruz Biotechnology. Antibodies for Phospho-NF-κB p65 (Ser536) (p-p65) and IκBα were from Cell Signaling Technology. The immunocomplexes were incubated with goat anti-rabbit or anti-mouse fluorescein-conjugated secondary antibodies, and then detected using an Odyssey fluorescence scanner (Li-Cor, Gene Company).

### Transient transfection

For plasmid transfection experiments, cells were transiently transfected using PEI (Polyplus; AFAQ) as described previously [24]. For RNA interference experiments, siRNA targeting Twist1 (si Twist1) or corresponding scrambled siRNA (si con) (Invitrogen) were transfected into cells using the INTERFERIN transfection reagent (Polyplus) according to the manufacturer’s protocol. Gene silencing effect was measured by Western blot assay at 60 h post transfection. The siRNA specific for Twist1 was designed with the sequence 5′-GAUGGCAAGCUGCAGCUAU-3′ as previously described [25].

### NF-κB Luciferase reporter assays

For the luciferase assay, cells were cotransfected with NF-κB promoter-luc and pRL-TK plasmids (Promega). After 24 h of transfection, cells were serum starved overnight followed by treated with or without TNFα (10 ng/mL) for additional 12 h, and the activities of firefly and Renilla luciferases were measured using a dual-luciferase reporter assay system (BioTek Synergy 2). Relative firefly luciferase activity was normalized by Renilla luciferase activity. Each point was set in duplication and the experiments were repeated three times.

### Cell proliferation assay

Cell proliferation was assessed by cell counts and Bromodeoxyuridine (BrdU) incorporation using a commercial kit (Millipore) according to the manufacturer’s instructions. Briefly, cells were serum free for 24 h. Then cells were trypsinized and equal number (5 × 10^3^) of cells from each group was plated into a 96-well plate and grown in complete culture medium with 10 μM BrdU for the indicated times. BrdU incorporation was detected by addition of peroxidase substrate. Spectrophotometric detection was performed at a wavelength of 450 nm using a microplate reader (BioTek Synergy 2). Each assay was done in triplicate and the experiments were repeated three times.

### Wound-healing, cell migration and invasion assay

Wound healing, Cell migration and invasion assays were performed as described previously [20]. Briefly, for wound-healing assays, monolayers of cells plated in 12-well plate were wounded by scraping with a 200 μL plastic pipette tip and then rinsed several times with medium to remove floating cells. The wound healing process was monitored at the indicated time with an inverted light microscope (Olympus IX70). For cell migration and invasion assays, Transwell filter chambers (Costar, Corning, NY) and BioCoat Matrigel invasion chambers (BD Biosciences) were used following the manufacturer’s instructions. Six random microscopic fields were counted per field in each group, and these experiments were repeated at least three independent times.

### In vivo metastasis and xenograft tumor assays

In vivo metastasis and xenograft tumor assays was performed as described previously [20]. For pulmonary metastatic model, five-week-old male nude mice, purchased from the Animal Center of The Second Military Medical University, were injected with 1 × 10^6^ cells from LM3-shcon, LM3-shA20, LM3-con or LM3-A20 through the tail vein, respectively. Mice were sacrificed at 3 months post injection. The lungs of each mouse were separated and fixed for H&E staining and lung metastatic foci were detected under microscope. For xenograft tumor model, nude mice were subcutaneously injected with the 2 × 10^5^ indicated cells into the bilateral flanks of each mouse, respectively. Tumor development was observed weekly or biweekly with a caliper, and the tumor volume was calculated using the following formula: larger diameter × (smaller diameter) ^2^ / 2. All animals were housed in cages under standard conditions, following the requirements of the Second Military Medical University Animal Care Facility and the National Institutes of Health guidelines.

### Statistical analysis

Data were presented as the means ± SEM unless otherwise indicated. Pearson chi-square test or student’s *t*-test was applied to assess the statistical significance among variables. Kaplan-Meier analysis with Log-rank test was used to compare the patient survival between subgroups. All statistical analyses were carried out using SPSS PASW Statistics 18.0 software (SPSS, Inc., Chicago, IL) and *p* value < 0.05 were considered to be statistically significant.

## Results

### Expression of A20 is increased in HCC tissues and cell lines

**Fig. 1:**
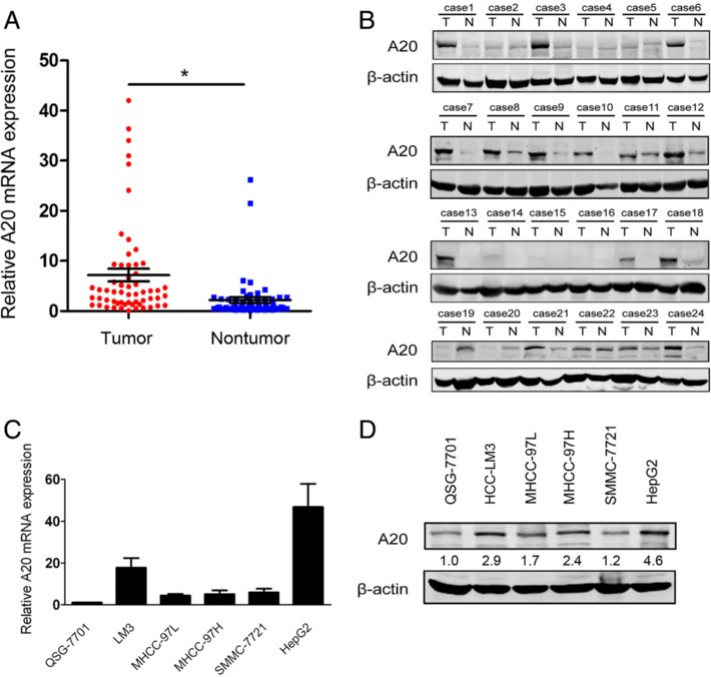
Expression of A20 is upregulated in HCC tissues and cell lines. **a** A20 mRNA expression in 60 paired human primary HCC tissues and matched adjacent non-tumor tissues were determined by real-time qPCR methods. Relative A20 mRNA expression results were normalized by internal control β-actin. *, *p* < 0.05. **b** Protein levels of A20 in an independent set of 24 paired HCC and matched adjacent non-tumor specimens were determined by Western blot assay. β-actin was used as a loading control. (T, tumor tissues; N, adjacent non-tumor tissues) (**c**-**d**) Expression levels of A20 mRNA (**c**) and protein (**d**) in QGS-7701 and HCC-derived cell lines

To determine the expression pattern of A20 in HCC, we first quantified the abundance of A20 mRNA in 60 pairs of HCC and corresponding adjacent non-tumor tissues using real-time qPCR methods. As showed in Fig. 1a, A20 mRNA expression was significantly increased in the tumor tissues compared with adjacent non-tumor tissues (*p* < 0.05). In addition, Western blot analysis from an independent set of 24 paired HCC and adjacent non-tumor specimens confirmed that A20 protein levels were significantly higher in cancerous tissues than in adjacent non-cancerous counterparts (Fig. 1b). Furthermore, we determined the levels of A20 mRNA and protein in HCC cell lines and the normal hepatocyte cell line QSG-7701. Similarly, A20 was significantly upregulated in all HCC cell lines when compared to the QSG-7701 cells at both the mRNA and protein levels (Fig. 1c and d). These results suggest that A20 expression is upregulated in HCC.

### Increased A20 expression predicts favorable clinicopathologic features and prognosis in HCC patients

**Fig. 2:**
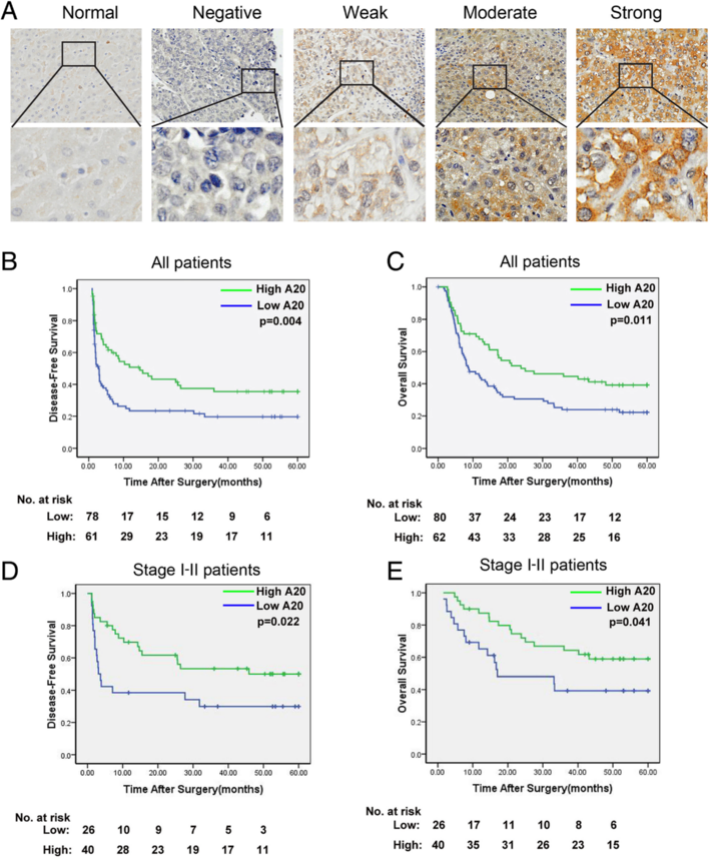
Upregulated A20 expression predicts favorable clinicopathologic features and prognosis in patients with HCC. **a** Representative immunohistochemical expression patterns of A20 in cancerous and adjacent normal mucosa specimens are shown. (Magnification, upper panel, ×100; lower panel, ×400) (**b**-**c**) Kaplan-Meier curves for disease-free survival (**b**) and overall survival (**c**) of all patients in the study cohort according to A20 expression status. The p-value was determined using the log-rank test. **d**-**e** Kaplan-Meier curves for disease-free survival (**d**) and overall survival (**e**) of patients with early stage (stages I-II) tumors according to A20 expression status. The p-value was determined using the log-rank test

To further determine the phenotypic expression of A20 protein in HCC clinical samples, immunohistochemical analysis was performed using a tissue microarray containing 143 pairs of HCC specimens. Each pair consisted of cancerous and adjacent non-cancerous tissues derived from the same patient. The representative staining of A20 protein (negative, weak, moderate, strong) in HCC tissues was shown in Fig. 2a, and positive staining of A20 was observed primarily in the cytoplasm. To evaluate the association between A20 expression levels and clinicopathologic characteristics, the patients were classified into high and low A20 expression subgroups with the median IRS value as the cut-off. As shown in Table 1, higher levels of A20 protein were significantly correlated with smaller tumor size (*p* = 0.007), lower TNM stage (*p* < 0.001), lower incidence of thrombus formation (*p* = 0.002), lower incidence of capsular invasion (*p* = 0.038), and lower levels of serum AFP (*p* = 0.013). While, there were no significant associations between A20 expression and sex (*p* = 0.847), patient age (*p* = 0.185), HBsAg (*p* = 0.078), Liver cirrhosis (*p* = 0.389) or capsular formation (*p* = 0.062).

Kaplan-Meier analysis of the complete study cohort indicated that patients in the high-A20 expression group had a significant prolonged disease-free survival (DFS, median DFS times were 8.6 and 2.4 months, respectively; difference = 6.2 months; *p* = 0.004, Fig. 2b) and favorable overall survival (OS, median OS times were 22.4 and 8.5 months, respectively; difference = 13.9 months; *p* = 0.011, Fig. 2c) than those in the low-A20 expression group. More importantly, stage-based survival analyses revealed that higher levels of A20 significantly predicted prolonged DFS (*p* = 0.022, Fig. 2d) and OS (*p* = 0.041, Fig. 2e) in patients who had early stage (stages I-II) tumors. These data indicate that the expression level of A20 could serve as a valuable indicator for predicting the prognosis of HCC.

### A20 inhibits the proliferation and tumor growth of HCC cells in vitro and in vivo

**Fig. 3:**
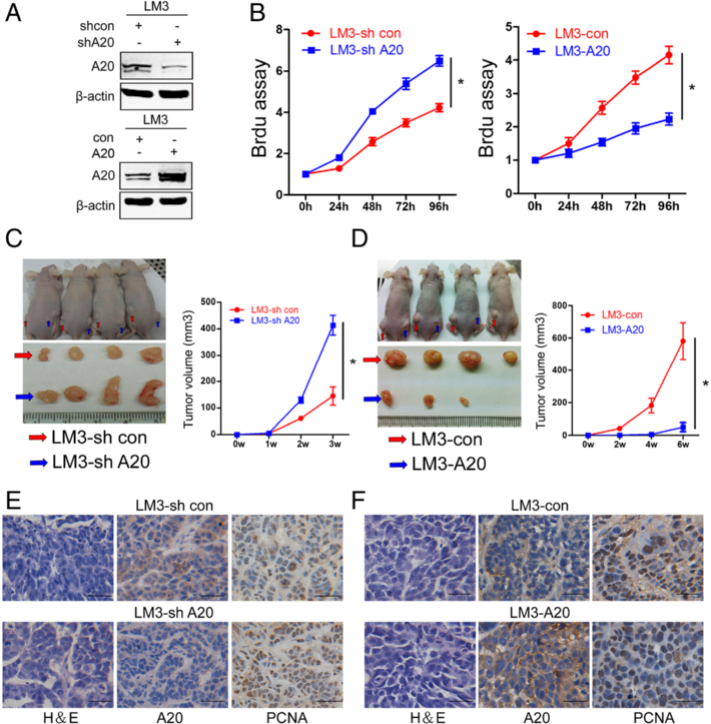
A20 suppresses proliferation and tumor growth of HCC cells in vitro and in vivo. **a** Stable knockdown or overexpression HCCLM3 cells (LM3-shA20 and LM3-A20, respectively) and their empty vector counterparts (LM3-shcon and LM3-con, respectively) were subjected to Western blot assay and probed with specific antibodies to A20 and β-actin. **b** Cell viability was determined by the Brdu cell proliferation assay at indicated times. Plots are mean ± SEM of data from three independent experiments. *, *p* < 0.05. **c** Xenograft tumor model assays. 2x10^5^ of LM3-shcon or LM3-shA20 cells were subcutaneously injected into the bilateral flanks of nude mice. Representative images of the subcutaneous xenografts are shown (left upper panel). Subcutaneous xenografts from the LM3-shcon and LM3-shA20 groups were excised from nude mice (*n* = 4) (left down panel). The growth curves of xenografts tumor volumes (right panel). *, *p* < 0.05. **d** 2 × 10^5^ of LM3-con or LM3-A20 cells were subcutaneously injected into the bilateral flanks of nude mice. Representative images of the subcutaneous xenografts are shown (left upper panel). Subcutaneous xenografts from the LM3-con and LM3-A20 groups were excised from nude mice (*n* = 4) (left down panel). The growth curves of xenografts tumor volumes (right panel). *, *p* < 0.05. **e**-**f** Indicated xenograft tumors were subjected to H&E and immunohistochemical staining of A20 and PCNA. Representative images are shown. The scale bars represent 50 μm

In view of the correlation between A20 expression levels and the aggressive clinical behaviors of HCC patients, it is speculated that A20 may play a role in HCC development and progression. To examine the effects of A20 on the proliferation of HCC cells, we established A20 overexpression or knockdown stable HCCLM3 cells (LM3-A20 and LM3-shA20, respectively) and their empty vector counterparts (LM3-con and LM3-shcon, respectively) (Fig. 3a). The cell proliferation assay showed that specific knockdown of A20 in HCCLM3 cells significantly increased cell proliferation, whereas overexpression of A20 in HCCLM3 cells did the opposite (Fig. 3b).

To further verify the growth-inhibitory effect of A20 in vivo, the xenograft tumor model assays were performed by subcutaneously injecting LM3-shA20 or LM3-shcon cells into the dorsal flank of nude mice. As shown in Fig. 3C, A20 depletion resulted in a significant increase in tumor growth (*p* < 0.05). Consistently, results from the xenograft experiments by injecting LM3-A20 or LM3-con cells into the dorsal flank of nude mice demonstrated that A20 overexpression significantly impaired the tumor growth in vivo (Fig. 3d). In addition, the xenograft tumors were subjected to immunohistochemical staining of proliferating cell nuclear antigen (PCNA), which is used as a proliferation marker. More PCNA-positive nuclei were observed in LM3-shA20 xenograft tumors than LM3-shcon xenograft counterparts (Fig. 3e). Conversely, the number of PCNA-positive nuclei was apparently decreased in the A20-overexpressing group as compared with the control group (Fig. 3f). These findings demonstrate that A20 plays a negative role in HCC proliferation.

### A20 suppresses the invasion and metastasis of HCC cells in vitro and in vivo

**Fig. 4:**
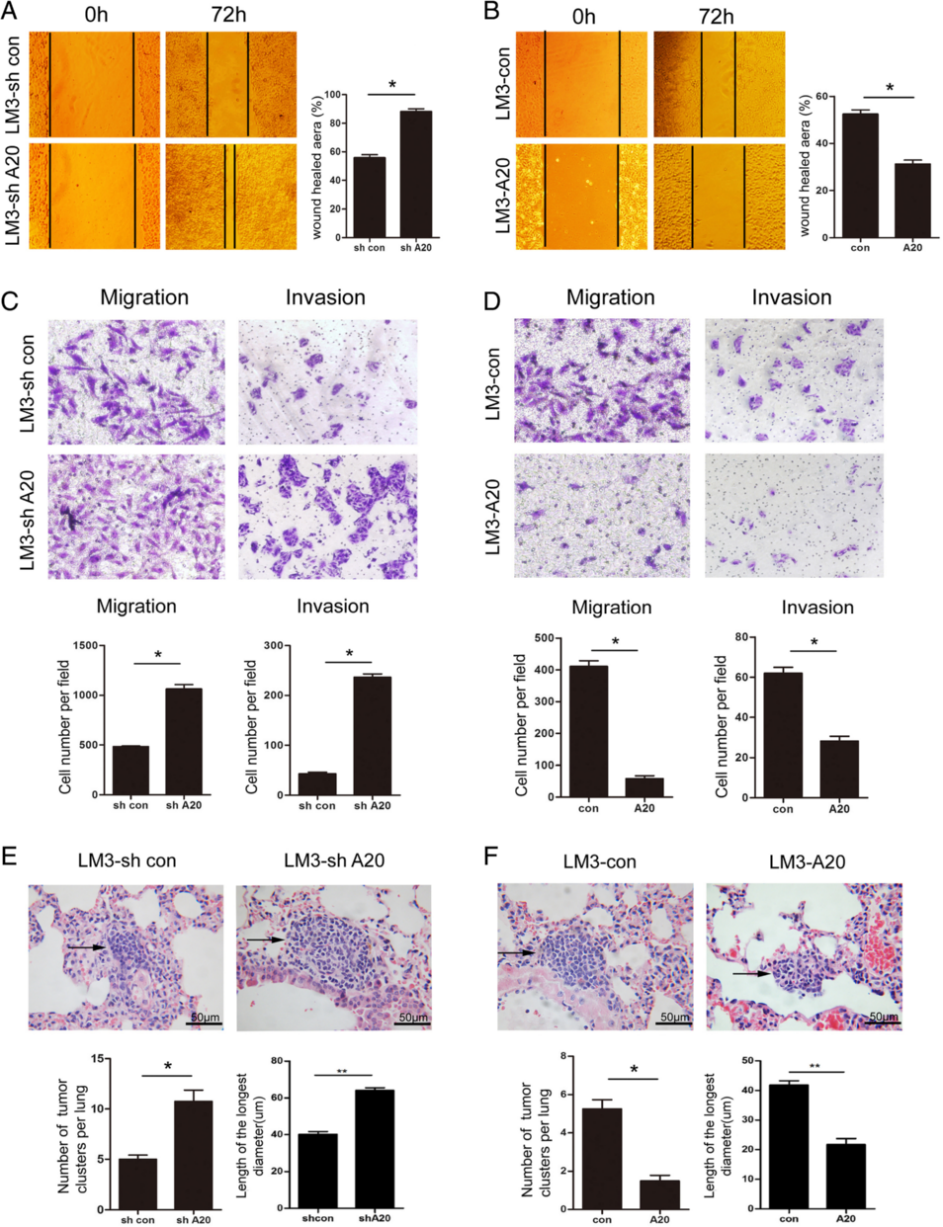
A20 inhibits the invasive and metastatic potential of HCC cells in vitro and in vivo. **a**-**b** Cells were plated in 12-well plate and allowed to grow to confluence. Medium was removed and wounds were introduced by scraping the confluent cell cultures with a 200 μL pipette tip. After treatment for 72 h, the wound healing process was monitored under an inverted light microscope. Representative results are shown. Magnification, ×100. Plots (right panel) are mean ± SEM of data from three independent experiments. *, *p* < 0.05. **c**-**d** Effects of A20 depletion (**c**) or A20 overexpression (**d**) on the migration and invasion of HCCLM3 cells were assessed by transwell migration assay and matrigel invasion assay, respectively. Representative results are shown in the left panel. Graphs below panels are numbers of migrated cells (mean ± SEM) from three independent experiments. *, *p* < 0.05. (**e**-**f**) LM3-shcon or LM3-shA20 cells (**e**) or LM3-con or LM3-A20 cells (**f**) were injected into the tail vein of nude mice (*n* = 5). Three months post inoculation, mice were sacrificed and metastatic tumor colonies in the lung were examined microscopically. Representative images of H&E staining of lung metastatic foci in each group are shown. The number and the length of metastatic nodules in the lungs of each group is measured and presented as the mean ± SEM below the panels. *, *p* < 0.05

To determine the roles of A20 in the motility of HCC cells, scratch wound healing, transwell migration and matrigel invasion assays were performed in A20 overexpression or knockdown HCCLM3 cells. In wound-healing assay, knockdown of A20 in HCCLM3 cells dramatically increased cell motility (Fig. 4a), while overexpression of A20 significantly inhibited this ability (Fig. 4b). In addition, the transwell migration assay showed that A20 depletion resulted in increased cell migration, whereas forced expression of A20 exerted the opposite effects. Moreover, similar results were also observed in the matrigel invasion assays (Fig. 4c and d).

To further confirm the metastasis-suppressing role of A20 in vivo, LM3-shA20 or LM3-shcon cells were injected into the lateral tail vein of nude mice. Three months later, more and larger micrometastatic lesions were detected microscopically in the lungs of nude mice inoculated with LM3-shA20 cells compared with those inoculated with LM3-shcon cells (Fig. 4e). By contrast, three months after LM3-A20 or LM3-con cells were injected into tail vein of nude mice, less and smaller micrometastatic lesions were observed in the lungs of nude mice inoculated with LM3-A20 cells than those inoculated with LM3-con cells (Fig. 4f). Collectively, these results indicate that A20 is capable of inhibiting the invasive and metastatic potential of HCC cells both in vitro and in vivo.

### Downregulation of Twist1 is required for A20-mediated inhibition of HCC proliferation and migration

**Fig. 5:**
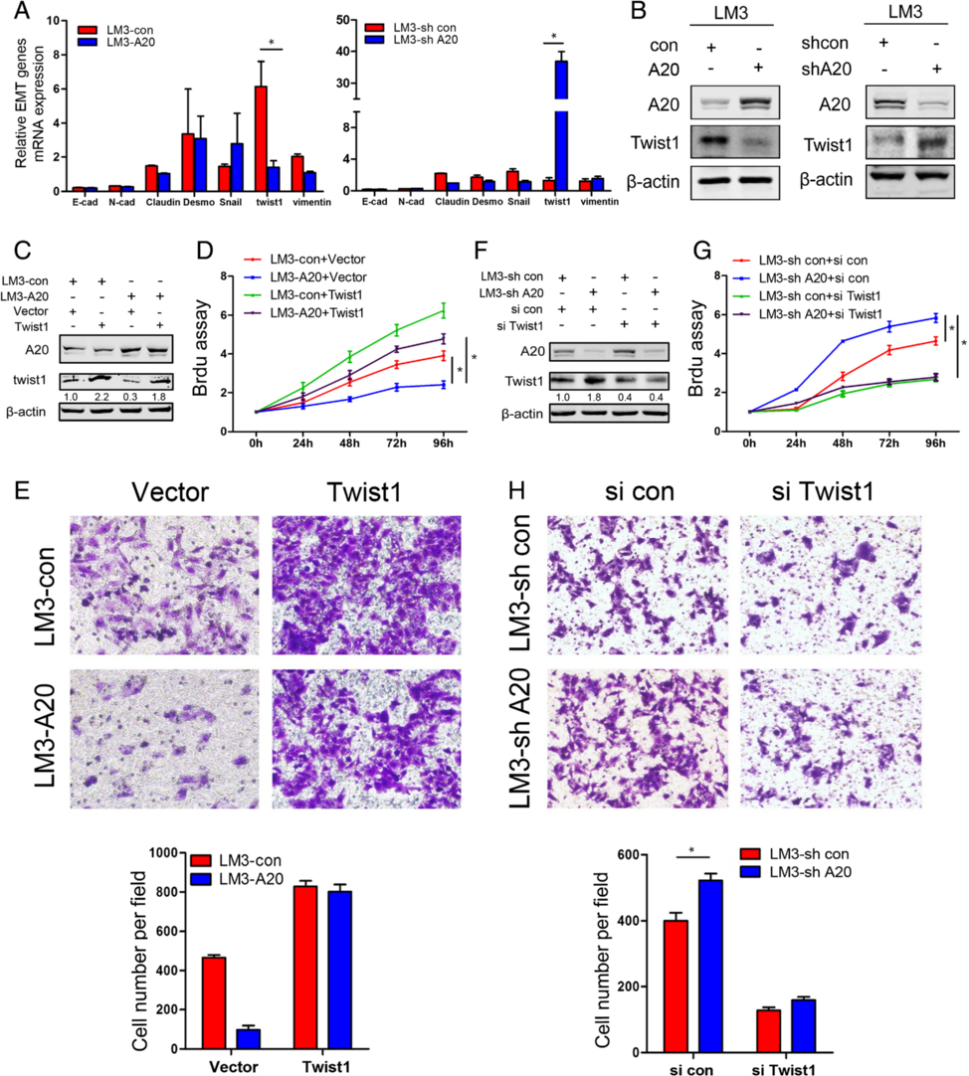
Downregulation of Twist1 is required for A20-mediated inhibition of proliferation and migration of HCC cells. **a** The total mRNA of indicated cells was extracted and the relative mRNA expression levels of EMT related genes were determined using qPCR methods. Results were normalized by β-actin. *, *p* < 0.05. **b** Protein levels of Twist1 in indicated cells were determined by Western blot assays. **c** Cells were transfected with pBabe-puro-flag-twist1 or empty vector for 24 h and then cell lysates were subjected to Western blot assay and probed with the indicated antibodies. **d** The proliferative properties of the cells treated as described for panel C were determined by the Brdu cell proliferation assay at indicated times. Plots are mean ± SEM of data from three independent experiments. *, *p* < 0.05. **e** The migratory properties of the cells treated as described for panel C were analyzed by transwell migration assay. Representative results are shown in the upper panel. Plots in the lower panel are mean ± SEM of data from three independent experiments. *, *p* < 0.05. **f** Cells were transfected with Twist1 siRNA (siTwist1, 20nM) or scrambled siRNA (si con, 20nM) for 60 h and then cell lysates were subjected to Western blot assay and probed with the indicated antibodies. **g** The proliferative properties of the cells treated as described for panel F were determined by the Brdu cell proliferation assay at indicated times. Plots are mean ± SEM of data from three independent experiments. *, *p* < 0.05. **h** The migratory properties of the cells treated as described for panel F were analyzed by transwell migration assay. Representative results are shown in the upper panel. Plots in the lower panel are mean ± SEM of data from three independent experiments. *, *p* < 0.05

To explore the underlying mechanism of A20-mediated inhibition of proliferation and migration of HCC cells, we assessed whether A20 is involved in the regulation of the epithelial-mesenchymal transition (EMT), which is thought to be a key process for cancer metastasis. Interestingly, among the EMT-related genes examined, Twist1 mRNA expression was found to be significantly decreased in LM3-A20 cells compared with LM3-con cells. Meanwhile, the Twist1 mRNA level was profoundly increased in LM3-shA20 cells when compared to control counterparts (Fig. 5a). In addition, western blot analysis confirmed that protein expression of Twist1 was significantly downregulated in LM3-A20 cells, while upregulated in LM3-shA20 cells when compared to their respective control counterparts (Fig. 5b).

To determine whether Twist1 is involved in A20-mediated regulation of proliferative and migratory capabilities, rescue experiments in HCCLM3 cells were performed. Importantly, ectopic expression of Twist1 effectively abrogated the A20-mediated inhibition of proliferation and migration in LM3-A20 cells (Fig. 5c, d and e). Conversely, specific knockdown of Twist1 expression by siRNA clearly blocked the increased cell proliferation and migration caused by A20 depletion in LM3-shA20 cells (Fig. 5f, g, h). Taken together, these findings suggest that A20-induced suppression of HCC proliferation and migration is mainly mediated through inhibition of Twist1 expression.

### Decreased NF-κB activity is involved in A20-induced downregulation of Twist1

**Fig. 6:**
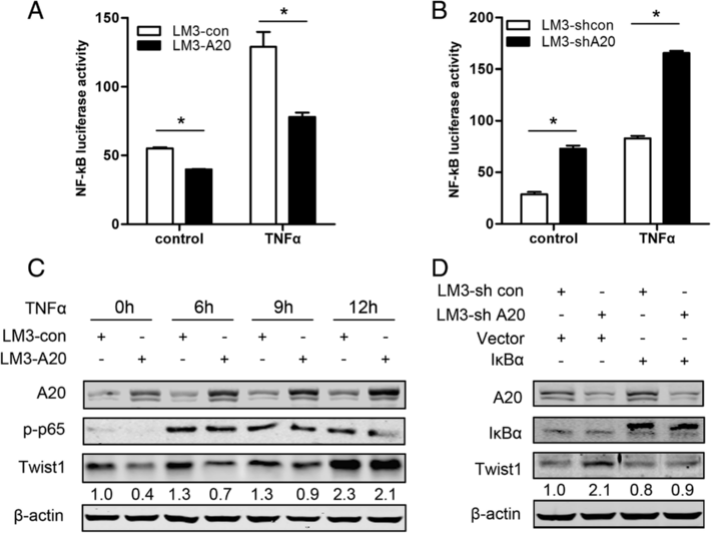
Decreased NF-κB activity is involved in A20-induced downregulation of Twist1. **a**-**b** Indicated cells were cotransfected with NF-κB promoter-luc (0.5ug/well) and pRL-TK plasmids (20 ng/well). After 24 h of transfection, cells were serum starved overnight followed by treated with or without TNFα (10 ng/mL) for additional 12 h, and cell lysates were prepared to perform a reporter gene assay. Plots are mean ± SEM of data from three independent experiments. *, *p* < 0.05. **c** Cells were serum starved overnight followed by treated with TNFα (10 ng/mL) for the indicated times and then cell lysates were harvested and subjected to Western blot assay and probed with the indicated antibodies. **d** Cells were transfected with IκBα or empty vector plasmid for 24 h and then cell lysates were harvested and subjected to Western blot assay and probed with the indicated antibodies

Previous studies have demonstrated that A20 negatively controls NF-κB signaling [26] and Twist1 could be regulated by NF-κB [27–29], we then determined whether NF-κB signaling contribute to reduced expression of Twist1 induced by A20. The effect of A20 expression on the transcriptional activity of NF-κB was determined by transient transfection assay using NF-κB-Luc reporter plasmid. As expected, the NF-κB luciferase activity was significantly decreased in LM3-A20 cells as compared with LM3-con cells either with or without TNFα treatment (Fig. 6a). Consistently, LM3-shA20 cells showed markedly increased NF-κB transcriptional activity when compared to control cells independent of TNFα treatment (Fig. 6b). Importantly, activation of NF-κB by TNFα treatment effectively rescued the reduced expression of Twist1 induced by A20 (Fig. 6c). Furthermore, enforced expression of IκBα, which profoundly inhibited the activity of NF-κB, blocked the upregulation of Twist1 expression in LM3-shA20 cells (Fig. 6d). These data indicate that suppression of Twist1 induced by A20 is mainly NF-κB dependent.

### Expression of A20 is inversely correlated with Twist1 levels and NF-κB activities in HCC tissues and cell lines

**Fig. 7:**
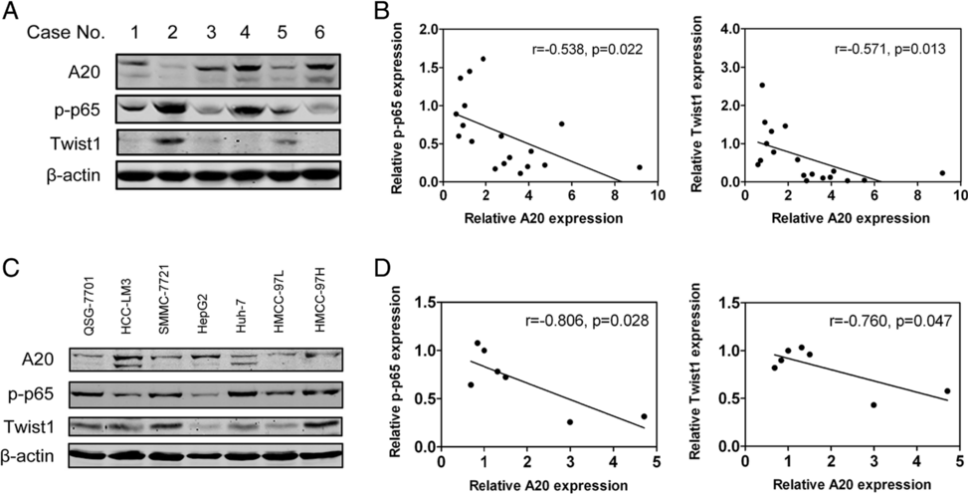
Expression of A20 is inversely correlated with Twist1 expression and NF-κB activities in HCC tissues and cell lines. **a** Protein levels of A20, p-p65, Twist1 and β-actin were determined by Western blots in 18 HCC samples. Representative results are shown. **b** The relative protein expression of A20, p-p65 and Twist1 in Fig. 7a was quantified and normalized to β-actin. Correlations between the relative expression of A20 and p-p65 or Twist1 in human HCC tissues were analyzed with the Pearson correlation formula. **c** Protein levels of A20, p-p65, Twist1 and β-actin in HCC cell lines were determined by Western blots. **d** The relative protein expression of A20, p-p65 and Twist1 in Fig. 7c was quantified and normalized to β-actin. Correlations between the relative expression of A20 and p-p65 or Twist1 in HCC cell lines were analyzed with the Pearson correlation formula

To further reveal the relationship between A20, activation of NF-κB and Twist1 in clinical HCC tissues, we performed Western blot analysis on protein levels of A20, p-p65 and Twist1 in human HCC specimens and cell lines (Fig. 7a and c). Levels of these proteins were normalized to each own level of β-actin. The relative density of each sample was used for the Pearson correlation analysis. The results showed that there were significantly inverse correlation between A20 and Twist 1, or A20 and p-p65 expression in both HCC tissues and cell lines (Fig. 7b and d). Collectively, these findings support that A20-induced effects on the proliferation and migration of HCC cells are mainly mediated through inverse regulation of Twist1 expression and the activation of NF-κB.

## Discussion

NF-kB has long been implicated in the development of inflammation and cancer [30]. Persistent activation of NF-κBin human tumors correlates with a higher incidence of metastasis, faster disease progression, increased tumor recurrence, poorer survival and therapeutic resistance [31]. Previous studies have revealed that activation of NF-kB signaling could promote HCC development by maintaining liver inflammatory responses [32–34]. Hence, inhibition of chronic liver inflammation by targeting the NF-kB signaling may have a great influence on the prevention of HCC development and progression. Recently, it has been clarified that A20 plays a primary role in suppressing pro-inflammatory signaling by attenuating NF-κB signaling via deubiquitylating RIPK1and TRAF6 [5, 35–37], which are crucial mediators of NF-κB activation. In addition, increased expression of A20 has been observed in HCC tissues [18]. Nevertheless, whether aberrant expression of A20 correlates with disease progression or plays a role in HCC has not been investigated.

The present study, to our knowledge, is the first to report the clinical implication and biological relevance of A20 in the development and progression of HCC. Using real-time qPCR and Western blot analysis, we found that A20 was upregulated in HCC tissues and HCC-derived cell lines at both the mRNA and protein levels. Our results are consistent with the previous finding of Dong BF et al. [18], in that they also reported that A20 was highly expressed in human HCC tissues. In addition, analyzing the association of A20 expression with clinicopathologic characteristics in 143 HCC patients by tissue microarray revealed a significant negative correlation between A20 expression and tumor size, thrombus formation, capsular invasion, TNM stage and serum AFP, which are all hallmarks for poor prognosis of HCC [38]. These findings suggest that A20 is highly expressed in a subset of human HCC which shows less aggressive tumor characteristics. As expected, Kaplan-Meier analysis indicated that patients with higher A20 expression levels had significant lower recurrence rates and longer survival times after curative resection. More importantly, stage-based survival analyses revealed that higher levels of A20 expression significantly predicted prolonged DFS and OS especially for patients who had early stage tumors. Generally speaking, HCC patients who had early stage tumors (stages I-II) have a favorable prognosis than those who had advanced stage tumors (stages III-IV). However, a subgroup of patients with early stage disease have an increased risk of early recurrence and death. Therefore, identification of this high-risk subgroup of patients would be of particular importance in the selection of patients for appropriate treatment. Thus, our results suggest that A20 protein expression status could be a attractive candidate marker to stratify patients with early stage HCCs into distinct risk subgroup and helps to guide individualized treatment.

The significant negative correlation between the expression level of A20 and the aggressive clinical behaviors and poor prognosis of HCC patients prompted us to investigate whether or not A20 plays a functional role in HCC development and progression. Indeed, our in vitro and in vivo experiments demonstrated that depletion of A20 enhanced the proliferative and invasive potential of HCC cells and triggered tumor growth and lung metastasis of HCC in nude mice, whereas enforced expression of A20 exerted the opposite effects. Thus, the results obtained from the gain-of-function and loss-of-function experiments clearly suggested a tumor-suppressor role of A20 in HCC, which was consistent with previous findings in lymphomas [7, 8, 39]. Interestingly, overexpression of A20 in the liver has been shown to promote hepatocyte proliferation to enhance liver regeneration [17]. Ferran C and his group previously found that A20 was significantly upregulated in the liver following partial hepatectomy and had the ability to protect mice against lethal radical hepatectomy by promoting hepatocyte proliferation through downregulation of the cyclin-dependent kinase inhibitor p21waf1 [40]. In addition, they recently further found that A20 induced liver regeneration following hepatectomy possibly by activating IL-6/STAT3 proliferative signaling through epigenetic downregulation of the negative regulator of IL-6 signaling and SOCS3 [41]. Moreover, the pro-tumorigenic function of A20 has also been revealed in several other solid tumors, such as inflammatory breast cancer [42], nasopharyngeal carcinoma, head and neck squamous cell carcinoma [43] and gliomas [13, 15]. In our study, knockdown of A20 clearly enhances the proliferative and invasive potential of HCC cells and triggers tumor growth and lung metastasis of HCC in nude mice, whereas overexpression of A20 exerted the opposite effects. Thus, the potential role of A20 as a tumor suppressor or tumor promoter is tissue type-specific and varies with the type of malignancy.

Twist1, the basic helix-loop-helix domain-containing transcription factor, is involved in the process of EMT and has been demonstrated to play a key role in the metastatic progression of human cancer [44, 45]. Interestingly, in the present work, we found that Twist1 was significantly downregulated in HCCLM3 cells exogenously expressing A20 while apparently upregulated in A20-depleted HCCLM3 cells. This observation encouraged us to further determine whether Twist1 is involved in mediating A20 action. Importantly, subsequent exogenous expression or knockdown experiments verified the requirement of downregulation of Twist1 expression for A20-induced suppression of HCC proliferation and migration. This finding was also in agreement with the well recognized pro-invasive and pro-proliferative effects of Twist1 on cancer progression [45–48]. Of note, although expression of Twist1 was significantly altered in A20-overexpressing or A20-depleting cells, no substantial changes in E-cadherin or N-cadherin expression or cellular morphological phenotype were observed in this setting, indicating that A20 may exert its anti-metastatic potential through a EMT-independent mechanism. In fact, recent studies have revealed that Twist1 contributes to tumor invasion and dissemination by regulating the expression of TIMP1 or MMPs [49–52]. Therefore, the detailed molecular events downstream of the repressed expression of Twist1 induced by A20 warrants to be further determined.

As Twist1 has been reported to be transcriptionally induced by NF-κB [27–29], we therefore hypothesized that NF-κB may be involved in the A20-induced downregulation of Twist1 expression. Indeed, by activation or inhibition of NF-κB signaling, our results confirmed that A20 suppresses Twist1 expression mainly through inhibition of NF-κB activation. Taken together, our current findings support the notion that A20 negatively regulates HCC cell proliferation and metastasis probably through inhibition of Twist1 expression via attenuating the NF-κB activity.

In conclusion, we reported here, for the first time, that upregulated A20 expression was significantly associated with less aggressive clinical behaviors and favorable postoperative outcome of HCC patients. In addition, A20 functions as a tumor suppressor in the progression and metastasis of HCC through a mechanism involving inhibition of Twist1 expression via suppressing NF-κB activation. Our findings encourage further investigation of its potential therapeutic implications in the treatment of patients with HCC.

## Additional file

Additional file 1: Table S1. Primers used in this study. (DOC 35 kb)

## Abbreviations

HCC: Hepatocellular carcinoma
OS: Overall survival
DFS: Disease-free survival
qPCR: Quantitative polymerase chain reaction
IHC: Immunohistochemistry
IRS: Immunoreactive score
NF-κB: Nuclear factor-κB
Twist1: Twist-related protein 1
TNM: Tumor-node-metastasis
