# Supplementary material for: A20 suppresses hepatocellular carcinoma proliferation and metastasis through inhibition of Twist1 expression
Source: Mol Cancer. 2015 Nov 4;14:186. doi: 10.1186/s12943-015-0454-6 (PMC4634191; doi:10.1186/s12943-015-0454-6)
Supplement: Additional file 1: Table S1. — Primers used in this study. (DOC 35 kb) [file 12943_2015_454_MOESM1_ESM.doc]

**Additional files**

**Additional file 1. Primers used in this study.**

| **Gene** | **Forward** | **Reverse** |
| --- | --- | --- |
| **A20** | 5’-CTCAACTGGTGTCGAGAAGTCC-3’ | 5’-TTCCTTGAGCGTGCTGAACAGC-3’ |
| **β-actin** | 5’-AATCGTGCGTGACATTAAGGAG-3’ | 5’-ACTGTGTTGGCGTACAGGTCTT-3’ |
| **Twist1** | 5’-GGAGTCCGCAGTCTTACGAG-3’ | 5’-TCTGGAGGACCTGGTAGAGG-3’ |
| **Claudin** | ,5’-ATCAGATGGGTATTCAAGAGGCG-3’ | 5’-ACTCCCACCGAGGTATGATGT-3’ |
| **Snail** | 5’-CTTCTCTAGGCCCTGGCTG-3’ | 5’-CATCTGAGTGGGTCTGGAGG-3’ |
| **Desmoplakin** | 5’-CAGGATGTACTATTCTCGGCG-3’ | 5’-ATCAAGCAGTCGGAGCAGTT-3’ |
| **Vimentin** | 5’-CGAAAACACCCTGCAATCTT-3’ | 5’-CTGGATTTCCTCTTCGTGGA-3’ |
| **E-cadherin** | 5’-CAGGTCTCCTCTTGGCTCTG-3’ | 5’-GACCGGTGCAATCTTCAAAA-3’ |
| **N-cadherin** | 5’-ACAGTGGCCACCTACAAAGG-3’ | 5’-CCGAGATGGGGTTGATAATG-3’ |
